# Monosodium urate crystals induce oxidative stress in human synoviocytes

**DOI:** 10.1186/s13075-016-1012-3

**Published:** 2016-05-21

**Authors:** Yessica Zamudio-Cuevas, Karina Martínez-Flores, Javier Fernández-Torres, Yahir A. Loissell-Baltazar, Daniel Medina-Luna, Ambar López-Macay, Javier Camacho-Galindo, Cristina Hernández-Díaz, Mónica G. Santamaría-Olmedo, Edgar Oliver López-Villegas, Francesca Oliviero, Anna Scanu, Jorge Francisco Cerna-Cortés, Marwin Gutierrez, Carlos Pineda, Alberto López-Reyes

**Affiliations:** Laboratorio de Líquido Sinovial, Instituto Nacional de Rehabilitación “Luis Guillermo Ibarra Ibarra”, Calzada México-Xochimilco 289, Tlalpan, 14389 Mexico City, Mexico; Laboratorio de Microbiología Molecular, Departamento de Microbiología, Escuela Nacional de Ciencias Biológicas (ENCB), Instituto Politécnico Nacional (IPN), Prolongación de Carpio y Plan de Ayala S/N Col. Casco de Santo Tomas, Miguel Hidalgo, 11340 Mexico City, Mexico; Biological and Health Sciences PhD program, Universidad Autónoma Metropolitana, Avenida San Rafael Atlixco 186, Iztapalapa, 09340 Mexico City, Mexico; Laboratorio Central de Microscopía, Departamento de Investigación, ENCB, IPN, Prolongación de Carpio y Plan de Ayala S/N Col. Santo Tomás, Miguel Hidalgo, 11340 Mexico City, Mexico; Rheumatology Unit, Department of Medicine-DIMED, University of Padova, Via Giustiniani, 2, Padova, 35128 Italy

**Keywords:** Monosodium urate crystals, Gout, Oxidative stress, Synoviocytes

## Abstract

**Background:**

Gout is the most common inflammatory arthropathy of metabolic origin and it is characterized by intense inflammation, the underlying mechanisms of which are unknown. The aim of this study was to evaluate the oxidative stress in human fibroblast-like synoviocytes (FLS) exposed to monosodium urate (MSU) crystals, which trigger an inflammatory process.

**Methods:**

Human FLS isolated from synovial tissue explants were stimulated with MSU crystals (75 μg/mL) for 24 h. Cellular viability was evaluated by crystal violet staining, apoptosis was assessed using Annexin V, and the cellular content of reactive oxygen species (ROS) and nitrogen species (RNS) (O_2_^-^, H_2_O_2_, NO) was assessed with image-based cytometry and fluorometric methods. In order to determine protein oxidation levels, protein carbonyls were detected through oxyblot analysis, and cell ultrastructural changes were assessed by transmission electron microscopy.

**Results:**

The viability of FLS exposed to MSU crystals decreased by 30 % (*P* < 0.05), while apoptosis increased by 42 % (*P* = 0.01). FLS stimulated with MSU crystals exhibited a 2.1-fold increase in H_2_O_2_ content and a 1.5-fold increase in O_2_^-^ and NO levels. Oxyblots revealed that the spots obtained from FLS protein lysates exposed to MSU crystals exhibited protein carbonyl immunoreactivity, which reflects the presence of oxidatively modified proteins. Concomitantly, MSU crystals triggered the induction of changes in the morphostructure of FLS, such as the thickening and discontinuity of the endoplasmic reticulum, and the formation of vacuoles and misfolded glycoproteins.

**Conclusions:**

Our results prove that MSU crystals induce the release of ROS and RNS in FLS, subsequently oxidizing proteins and altering the cellular oxidative state of the endoplasmic reticulum, which results in FLS apoptosis.

**Electronic supplementary material:**

The online version of this article (doi:10.1186/s13075-016-1012-3) contains supplementary material, which is available to authorized users.

## Background

Gout is a uric acid (UA) metabolic disorder that promotes the formation and deposition of monosodium urate (MSU) crystals inside joints and periarticular soft tissues as a result of hyperuricemia. It is the most common inflammatory arthropathy in young men, and its prevalence is underestimated due to the long asymptomatic phase of the disease [1]. The global burden of gout is substantial and has increased in many parts of the world over the past 50 years [2]. Gout causes monocytic inflammatory cells to phagocytose MSU crystals. This induces the release of pro-inflammatory cytokines such as IL-8, IL-6, CCL2, interferon (IFN)-γ, and IL-1β by assembling and activating the NOD-like receptor pyrin containing 3 (NLRP3) inflammasome [3, 4].

The deleterious effects of urate are primarily attributed to its ability to trigger the formation of reactive oxygen species (ROS) and activate NLRP3. However, these mechanisms have not yet been elucidated [5, 6]. The activation of NADPH oxidase, xanthine oxidase, and nitric oxide synthase enzymes generates hydrogen peroxide (H_2_O_2_), superoxide anion (O_2_^-^) and nitric oxide (NO), respectively. The interaction of these last two molecules promotes the generation of peroxynitrite (ONOO^-^), which in turn increases apoptosis, the degradation of connective tissues, and joint damage [7, 8]. However, due to the complex interactions that take place within joints among various cell types, including neutrophils, macrophages, mast cells, endothelial cells and synovial fibroblasts, it is possible for synovial fibroblasts to play a role in modulating the inflammatory response to MSU crystals in patients with gout [9, 10].

Previous studies have reported that endogenous ROS are overproduced during acute gout attacks, suggesting that oxidative stress (OS) contributes to acute gout attacks and to the painful and inflammatory responses that MSU crystals induce by currently unknown mechanisms [11]. The aim of this study was to evaluate the pro-oxidizing effect of MSU crystals in an in vitro model of crystal-induced inflammation. We focused on ROS and RNS associated with the generation of OS induced by MSU crystals in human-derived synovial membrane (SM) cells. We found that MSU crystals trigger an oxidative response and oxidize proteins, highlighting a possible mechanism underlying gout pathogenesis. The results shown here help explain how MSU crystals combined with ROS react with proteins of synoviocytes, increasing our understanding of the role of OS in the development of gout.

## Methods

This study was approved by the Research Committee of the Instituto Nacional de Rehabilitacion (Ref.02/13) of Mexico and was carried out according to the principles of the Helsinki declaration. Written informed consent was obtained from all patients.

### MSU preparation

MSU crystals were synthesized by uric acid (UA) crystallization according to the method described by Denko and Whitehouse [12] and modified by Scanu et al. [10]. MSU crystals were characterized by polarized light microscopy and scanning electron microscopy (SEM) based on the crystallographic characteristics birefringence, size, and morphology [13], and were sterilized at 180 ° C for 2 h. The absence of microbial contaminants was confirmed by culturing for microorganisms, and the crystals were determined to be bacterial endotoxin-free by Limulus amebocyte cell-lysate assay (Sigma-Aldrich).

### Isolation and cell culture of fibro-synoviocytes

A primary culture of synoviocytes was obtained via mechanic-enzymatic breakdown of SM collected from patients with osteoarthritis (OA) (*n* = 5) during knee joint replacement. Synoviocytes were isolated from tissue explants following digestion with collagenase type IA (1 mg/mL) (Gibco, Life Technologies) for 2 h with mixing at 37 °C. Cells were seeded in T25 flasks at a density of 250,000/flask until confluence. The cells were cultured in DMEM-F12 supplemented with 10 % fetal bovine serum and 1 % penicillin-streptomycin (Gibco, Life Technologies), and they were incubated in a controlled CO_2_ atmosphere at a regulated temperature. At confluence, cells were harvested (TrypLE Express, Gibco, Life Technologies) and seeded into new flasks that kept synoviocytes from different patients separated. For the experiments, cells were used at the third or fourth passage.

### Phenotyping of fibroblast-like synoviocytes analysis

#### Characterization by qRT-PCR

Upon the third passage, fibroblast-like synoviocytes (FLS) phenotype was determined by assessing the expression of the uridine diphosphate glucose dehydrogenase gene (*UGDH*), and *CD14* gene was used for macrophage-like synoviocytes by qRT-PCR. Total RNA from each patient was extracted by the Trizol method [14]. The qRT-PCR technique was performed by amplifying primers (Additional file 1) in a Rotor-Gene Q thermocycler (Qiagen), according to the commercial kit RT^2^ First Strand Kit from Qiagen. The results were normalized to the housekeeping *GAPDH* gene and relative quantification was performed through REST-09 software (Relative Expression Tool software 2009). After amplification, a melting assay was performed to confirm the specific size of the products of each gene.

#### Characterization by immunofluorescence and western blot

Expression of prolyl-4-hydroxylase (PDH4) was evaluated by immunofluorescence assay (IFA) and Western blot (WB). For IFA, cells were seeded into fixed and permeated chamber-slides. Subsequently, primary antibody PDH4 (ab108980, Abcam) was incubated. Afterwards, secondary antibody (ab175471 Alexa Fluor® 568, Abcam) was incubated. Finally, images were captured with an Ism 5 beta Carl Zeiss microscope.

Total protein was obtained from the culture of sinoviocytes. Analysis of the protein content was performed by WB according to Serratos et al. [15] Normalization was performed with Beta-actin antibody from Sigma (A3854). Blots were revealed using Immobilon Western Chemiluminescent HRP Substrate (Millipore Corporation, USA). The blots were scanned with an Amersham Imager 600 RGB (GE) and densitometry was analyzed using ImageQuant TL 8.1 software.

#### Characterization by flow cytometry

To evaluate surface markers associated with fibroblasts and macrophage, a flow cytometry (FC) assay was performed according to Landa-Solís C et al. [16] Cells were marked with monoclonal antibodies PE-conjugated CD166 and PE-conjugated CD14 from BD PharMigenTM (San Diego, CA, USA). Data were collected through a BD FACSCalibur flow cytometer and analyzed with CellQuestTM PRO software (Becton-Dickinson).

### Cell stimulation, viability, and apoptosis

FLS were treated for 24 h with MSU crystals at 0, 60, 75, 80, and 100 μg/mL. Cell viability was assessed by the crystal violet method [17] after MSU crystal cell stimulation. Based on these results, only one concentration was used for all subsequent tests. FLS apoptosis was assessed by FC detection of annexin V using a commercial kit (Annexin V Alexa Fluor 488 from Molecular Probes). Treatment with 100 μM H_2_O_2_ for 30 minutes was used as positive control for oxidation because an increase in H_2_O_2_ formation is associated with inflammation and fast OS induction in cells [18]. Unstimulated cells were used as negative control.

### Assessment of oxidative stress

Oxidative stress was evaluated by determining intracellular O_2_^-^ through oxidation of dihydroethidium (DHE, hydroethidine) at a 606 nm emission wavelength, according to the manufacturer’s instructions, using a Tali Image-based Cytometer (Life Technologies). H_2_O_2_ was detected by oxidation of 5-, 6- carboxy-2′, 2′, 7′-diclorofluorescein diacetate (carboxy-H2DCFDA) (Image-iT LIVE Green Reactive Oxygen Species Detection) using a fluorescence reader (BD, Beckman Coulter, AXT-800) at 530 nm. NO was quantified by benzotriazole formation with a commercial kit, DAF-FM (4-amino-5-methylamino-2,7-difluorofluorescein diacetate, Molecular Probes) at 515 nm in Tali Image-based Cytometer. Data analysis was performed based on fluorescence intensities.

### Protein oxidation

After derivatization using 2, 4-dinitrophenylhydrazine (DNPH), the protein oxidation products were identified by scanning carbonyl groups with the OxyblotTM Protein Oxidation Detection Kit (Millipore Inc.) according to the manufacturer’s instructions. Image detection was performed with two methods: a conventional chemiluminescent detection and a fluorescence method using ECL Plex goat-alfa-rabbit IgG-Cy5 (GE, Healthcare), a 630 nm excitation filter and a 670 nm emission filter. The images were scanned with Amersham Imager 600 RGB (GE, Healthcare), and analyzed using ImageQuant TL 8.1 software.

### Morphostructural characterization by transmission electron microscopy

The FLS were fixed with 2.5 % glutaraldehyde, treated with 1 % osmium tetroxide, and dehydrated with alcohol and propylene oxide. The samples were embedded in an epoxy resin and polymerized at 60 °C for 24 h. Sections were cut 80–90 nm thick, and stained with 4 % uranyl acetate and lead citrate. The cells were then analyzed under a transmission electron microscope (TEM; Philips, model Tecnai 10) equipped with a Mega View II digital camera. A voltage of 80 kV was employed.

### Statistical analysis

Each experiment was performed at least three times with the sample from each patient in independent experiments. Mean values were statistically analyzed with GraphPad Prism v. 6.0 using variance analysis, followed by the one-way post hoc Dunnett test. *P* < 0.05 was considered statistically significant.

## Results

### MSU crystallization

As evidenced by polarized light microscopy and SEM, chemically synthesized MSU crystals exhibited a characteristic needle-shaped negative birefringence, a 5–40 μm range size, and triclinic structure [19]. In addition, two blinded experts reported similar morphological characteristics in synthetic MSU crystals and those obtained from the synovial fluid of patients during an acute gouty attack, and they were unable to differentiate between the two types of crystals (data not shown) (see Additional file 2).

### FLS characterization

FLS isolated during SM biopsies expressed the PDH4 protein, *UDGH* gene and CD166 membrane receptor, as assessed by WB, IFA, qRT-PCR and FC, respectively (data not shown) (see Additional files 3, 4, and 5).

### Assessment of cell viability and apoptosis

**Fig. 1:**
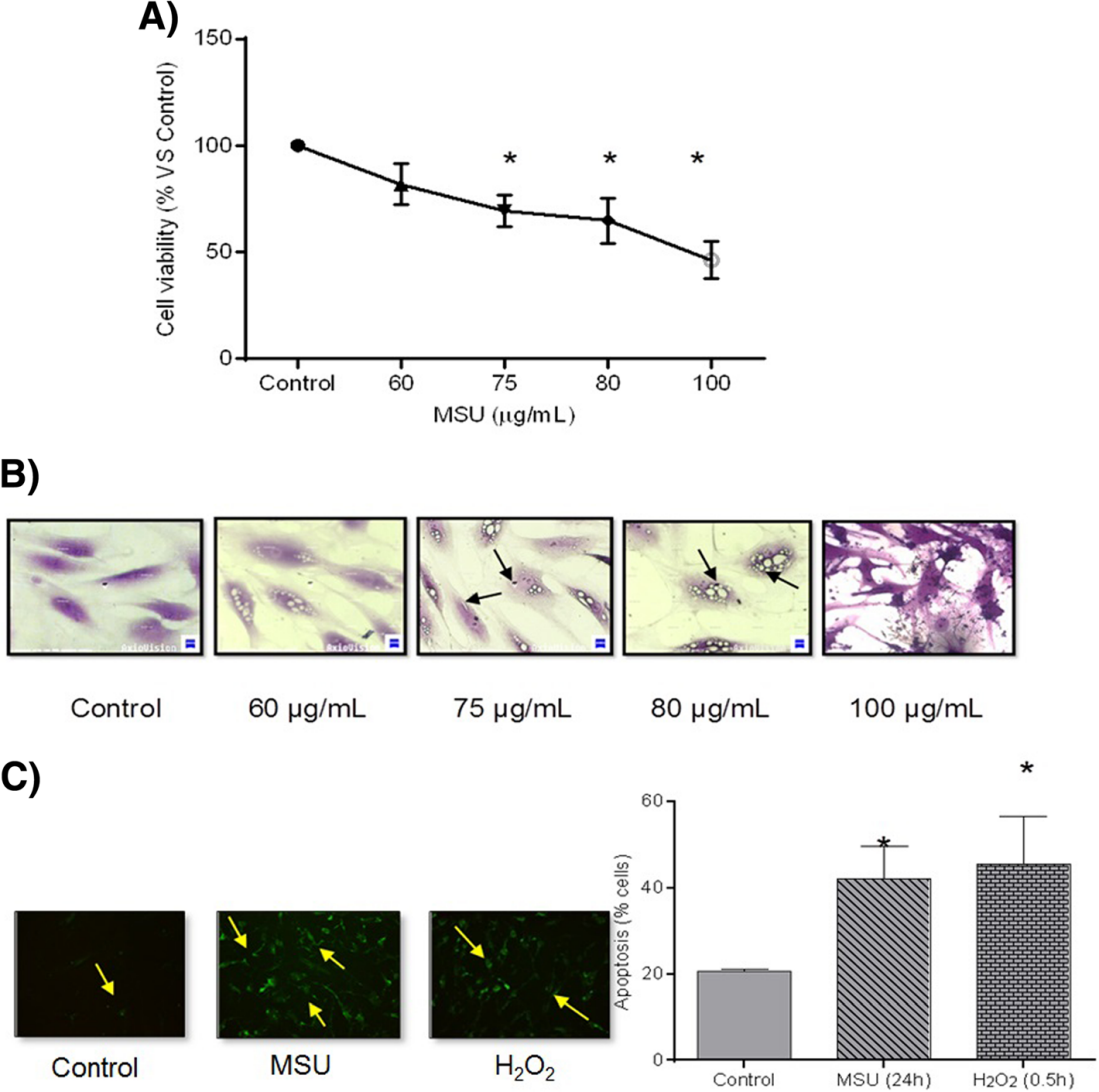
Cellular response to the presence of monosodium urate (*MSU*) crystals. **a** Cell viability after a 24 h treatment. **b** Cell morphological changes after MSU crystal exposure. The arrows indicate the intracellular vacuoles of MSU crystals. **c** Apoptosis is revealed by Annexin V detection (*yellow arrows*) in synoviocytes exposed to MSU crystals and H_2_O_2_ (100 μM). Additionally, columns show quantification of the apoptotic cells by flow cytometry. Values are expressed as the mean ± standard deviation *P < 0.05 vs control

Cell cultures exposed to MSU crystals at a concentration of 75 μg/mL maintained a viability of 77 % ± 0.50, while a concentration of 100 μg/mL resulted in a viability of 54.82 % ± 0.46 compared to unstimulated FLS (*P* ≤ 0.05) (Fig. 1a). FLS exposed to 75 μg/mL MSU had invagination of crystals in the cytoplasm and cellular stress (Fig. 1b).

Adding MSU crystals at 75 μg/mL or H_2_O_2_ at 100 μM induced apoptosis in 42 and 45 % of cells, respectively, compared to 21 % cell apoptosis observed in unstimulated cultures. This increment was significant (*P* ≤ 0.05) (Fig. 1c). Based on these results, we used a concentration of 75 μg/mL MSU for all subsequent experiments.

### Evaluation of oxidative stress

**Fig. 2:**
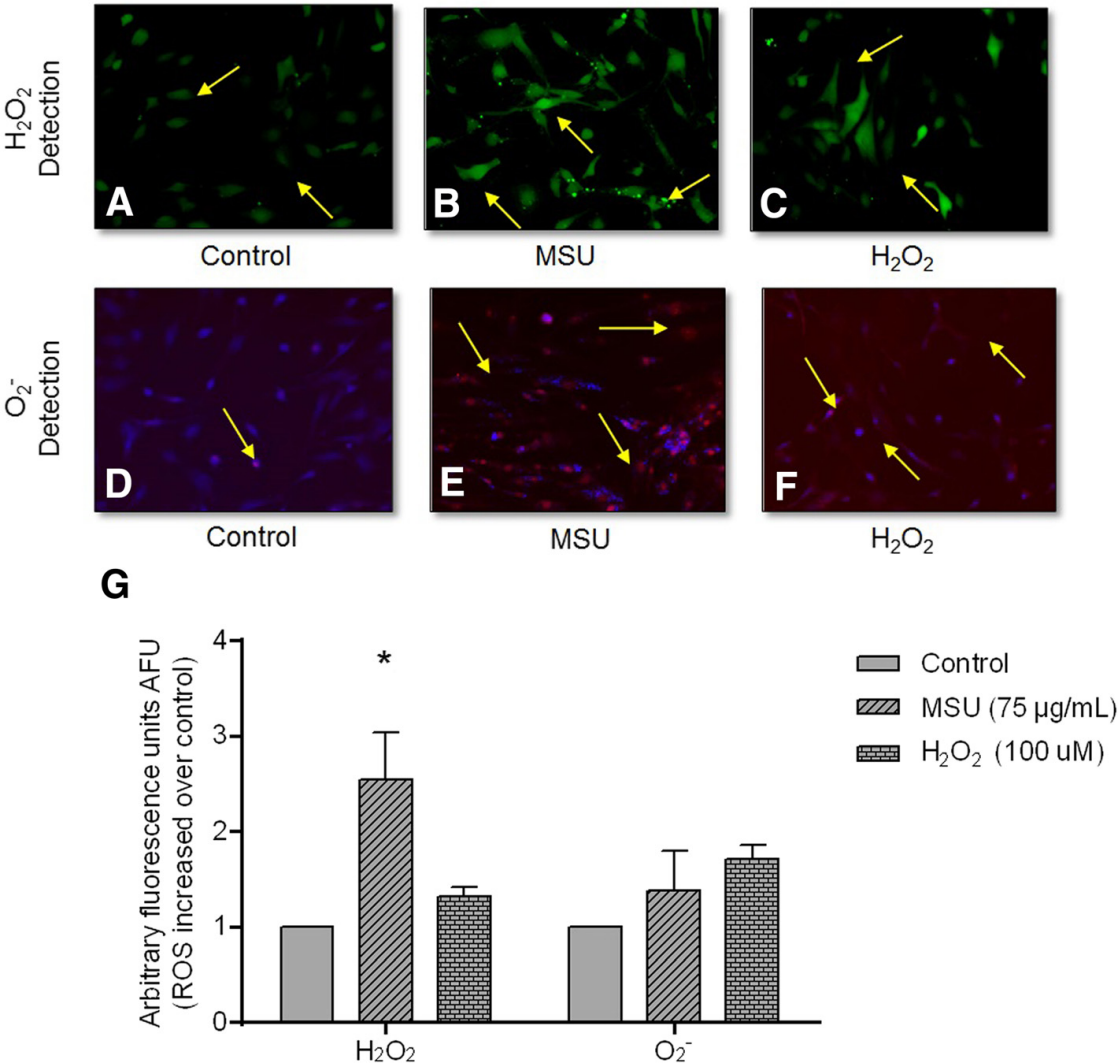
Monosodium urate (*MSU*) crystals increase reactive oxygen species (ROS) in synoviocytes. Arrows indicate intracellular H_2_O_2_ formation, which is revealed by DCFH oxidation (*green fluorescence*) in untreated fibroblast-like synoviocytes (FLS) (**a**); FLS treated with MSU crystals at 24 h (**b**), and FLS treated with H_2_O_2_ at 30 minutes (**c**). *Arrow*s indicate O_2_^-^ intracellular production by oxidation of dihydroethidium (DHE) (*red fluorescence*) in untreated FLS (**d**); FLS treated with MSU crystals (**e**), and FLS treated with H_2_O_2_ (**f**). *Bars* show quantification of DCFH and DHE fluorescence: data are reported as units of arbitrary fluorescence (*UAF*) (**g**). Values are expressed as the mean ± standard deviation; *P < 0.05 vs control

**Fig. 3:**
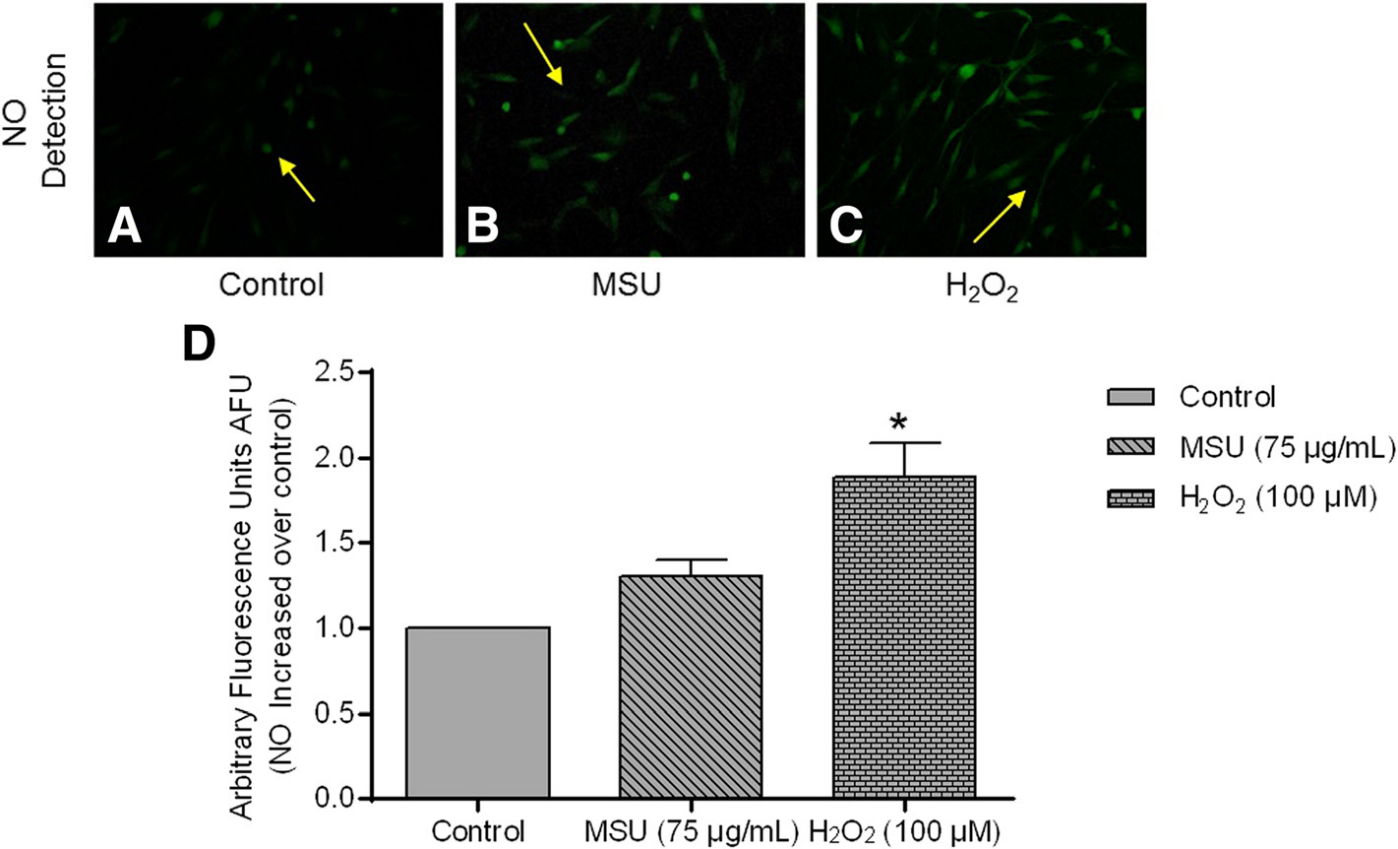
Nitric oxide (NO) production in synoviocytes. **a** Detection of NO in untreated fibroblast-like synoviocytes (FLS). **b** FLS treated with monosodium urate (*MSU*) crystals. **c** FLS treated with H_2_O_2_. Arrows indicate fluorescence produced by intracellular NO. **d** Bars show NO quantification by Tali image-based cytometer. Values are expressed as the mean ± standard deviation; *P < 0.05 vs control

MSU crystals induced a 2.5-fold increase in intracellular production of H_2_O_2_ in comparison to untreated FLS (*P* < 0.05), and a 2.1-fold increase compared to a positive control (Fig. 2a-c and g). Similarly, stimulating FLS with crystals for 24 h or with H_2_O_2_ for 30 minutes yielded 1.5-fold and 1.8-fold increases in O_2_^-^, respectively, compared to untreated FLS (Fig. 2d-f and g). Finally, only a 1.5-fold increase in NO was observed in FLS upon exposure to MSU crystals (Fig. 3).

### Analysis of oxidized proteins

**Fig. 4:**
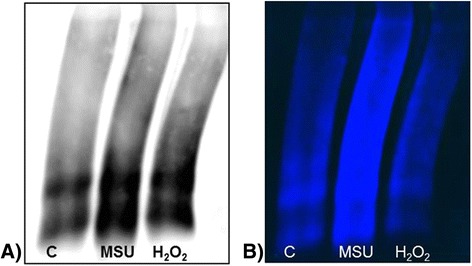
Oxidized proteins assay. **a** Representative oxyblot of fibroblast-like synoviocytes (FLS) proteins from control group (*line 1*); FLS proteins exposed to monosodium urate (*MSU*) crystals (*line 2*); and FLS proteins from the positive control sample (*line 3*). **b** Oxidation scan with fluorescence detector. Results are representative of independent experiments with cells from different patients

FLS exposed to MSU crystals had a protein oxidation pattern similar to the one observed in H_2_O_2_-treated cells, in comparison to unstimulated FLS (Fig. 4a, b).

### Ultrastructural analysis

**Fig. 5:**
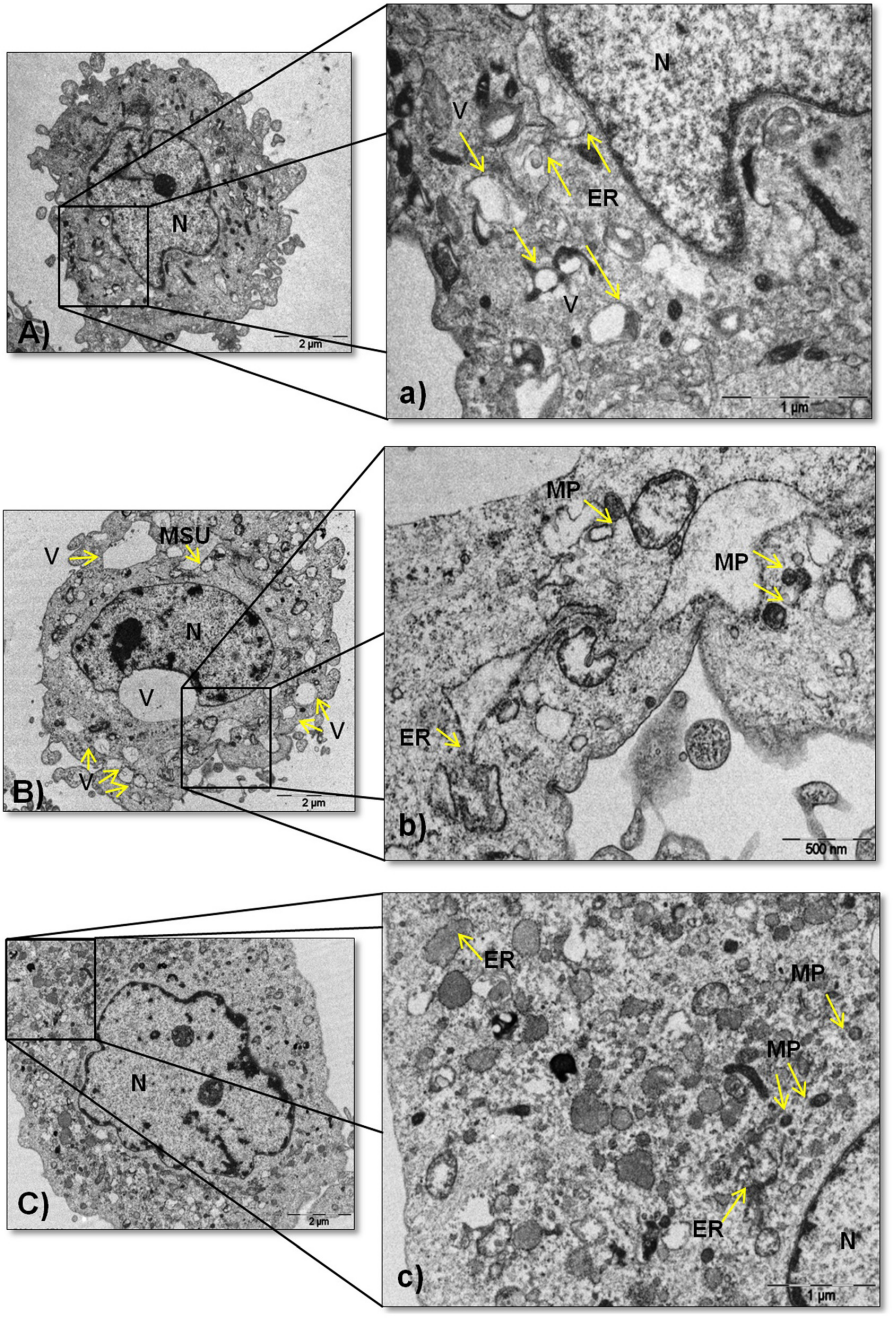
Ultrastructural changes in synoviocytes. **A** Ultrastructure of an untreated fibroblast-like synoviocytes (FLS). **a** Magnified view of the section is indicated by a *black box* showing the nucleus (*N*), endoplasmic reticulum (*ER*) and vacuoles (*V*) highlighted with arrows. **B** FLS treated with monosodium urate (*MSU*) crystals at 75 μg/mL exhibiting N, swollen vesicular structures of different sizes, and MSU crystal cavity. **b** A high-magnification image showing misfolded proteins (*MP*) aggregates and ER indicated with *arrows*. **C** FLS treated with H_2_O_2_ at 100 μM showing N. **c** A magnified view of the section is indicated by a *black box* showing MP aggregates, ER and N. Results are representative of one of five separate experiments with FLS from different patients

Untreated FLS were analyzed under transmission electron microscopy (TEM). The cells exhibited irregular nuclei containing loose chromatin; the mitochondria, rough endoplasmic reticulum (ER) and vacuoles were distributed homogeneously throughout the cytoplasm; and cytoplasmic prolongations were observed (Fig. 5a, b).

In addition to the presence of intracellular crystals, MSU crystal-stimulated FLS had an increased number of vacuoles and a reduction of ER (Fig. 5c). Furthermore, aggregates of misfolded glycoproteins (MP) were evident in the lumen (Fig. 5d). These aggregates were also observed to a lesser extent in the FLS incubated with H_2_O_2_, although few cytoplasmic prolongations were present (Fig. 5e, f).

## Discussion

The current study revealed that MSU crystals are able to decrease cell viability through apoptosis induction in FLS. Although the definitive mechanism for MSU-induced apoptosis has not been established, it has been demonstrated that MSU crystals exert different apoptotic effects depending on the cell type interacting with the crystals. While some studies have reported that MSU crystals inhibit neutrophil apoptosis [20], others have shown that they do not induce any change in the percentage of apoptosis for osteoblast-like cells [21]. Recently, MSU crystals have been shown to promote renal cell apoptosis through a mechanism involving ROS generation [22]. However, no data were available on their influence on FLS. According to one report, apoptosis is induced in chondrocytes isolated from patients with RA following stimulation with MSU crystals [23]. The association of this apoptotic state with the loss of cartilage repair and regeneration capacity could highlight a link between FLS apoptosis and the tissue damage observed in gouty patients. Moreover, the relationship between the increment of ROS and NO and the loss of FLS viability caused by MSU crystals is consistent with published findings [24–26].

In addition, we established that crystal-exposed FLS produced H_2_O_2_, O_2_^-^ and, to a lesser extent, NO, promoting a state of cellular oxidation. One mechanism involved in ROS production is the NADPH oxidase system in THP-1 cells stimulated with MSU crystals [27]. This mechanism of ROS generation has also been shown in FLS from patients with OA and RA that were exposed to TNF-α and IL-1β, exhibiting a heightened state of cellular oxidation [28]. Our experiments proved, via an increase in ROS/RNS, that MSU crystals activated an oxidative state in FLS. The increase in H_2_O_2_ observed in FLS exposed to MSU crystals for 24 h is similar to that reported for FLS stimulated with advanced oxidation proteins products; a threefold to eightfold increase in H_2_O_2_ was observed compared to unstimulated control cells [29]. This suggests that MSU crystal-mediated ROS overproduction in FLS is involved in the disturbance of homeostasis within the joint microenvironment, which can damage all cellular components, including DNA, lipids and proteins [30]. However, proteins are possibly the most immediate vehicle for inflicting oxidative damage on cells because they are often catalysts. Therefore, we assessed the influence of ROS in oxidized protein content of FLS affected by MSU crystals.

The impact of ROS on the proteins of FLS with MSU crystals was clearly seen on images because there were more spots and with higher intensities than in control cells, indicating increased carbonyl content. While there are no reports that can be directly compared to our data, accumulation of protein carbonyls [31] has been observed in some rheumatic diseases (including RA and psoriasis), but it is known that exposure of proteins to ROS leads to denaturalization, loss of function, crosslinking, aggregation, and fragmentation. Under these conditions, it is suggested that accumulation of some compounds in the joint, like glycosaminoglycans and hyaluronic acid, cause damage by reducing joint viscosity [32]. However, there are no studies of the underlying mechanism. We suspected that the increase in OS might be contributing to synovial cell damage altering the functional and structural integrity. Therefore, we visualized OS-induced ultrastructural changes triggered by MSU-crystals in gout. In our model, we observed an increase in rough ER and in the presence of MP aggregates due to cellular stress in the FLS. These findings are similar to those described for synoviocytes exposed to an adjuvant used for treating arthritis (i.e., a reduction of the Golgi apparatus, mitochondria and ER [33]), and to the ones describing the appearance of vacuoles in FLS cytoplasm due to the internalization of particles. In addition, intracellular lysosomes and other cytoplasmatic formations were found [34], and these morphological changes suggest the induction of autophagy in the cells [35].

An important unanswered question is the mechanism responsible for activating OS in response to MSU crystals in FLS. We can speculate that this effect might be related to the mechanism involved in the overproduction of ROS and the decrease of anti-oxidative enzymes caused by lead-induced OS [36]. However, the molecular pathways involved in MSU-induced OS in FLS are not yet completely understood. Inhibition studies of these pathways may be helpful to understand the signaling network behind MSU crystals.

## Conclusions

In conclusion, this study reveals that the exposure of FLS to MSU crystals promotes an oxidizing state, which may induce an apoptotic state and decrease cell viability and synovial integrity. Nevertheless, further studies are needed to achieve a better understanding of the signal transduction pathways by which MSU crystals enhance the damage generated in FLS, and to elucidate the molecular mechanism of OS in gout. This study confirms the oxidative role of MSU crystals in FLS, which could contribute to the inflammation and pain experienced during an acute gout attack. This model of OS in FLS is important for determining the role of antioxidants involved in local and systemic damage to the joint in the development of novel therapeutic targets to block OS.

## Additional files

Additional file 1: Table S1. Primers used for qRT-PCR gene analysis. (DOCX 14 kb) Additional file 2: Synthetic crystals of monosodium urate. A Scanning electron micrograph of synthetic MSU crystals (×50,000). B MSU crystals under polarized light (×400). C MSU crystals from the synovial fluid of a gout patient (×400). The *white bar* indicates the axis compensator. (DOCX 1750 kb) Additional file 3: Characterization of synoviocytes derived from five primary cultures from different patients. A Typical morphology of fibroblasts. B Expression of PDH4+ in synoviocytes by IFA, expression of intracellularly localized PDH4+ by confocal microscopy. C Protein expression of PDH4+ by WB. (DOCX 840 kb) Additional file 4: UGDH gene expression and CD14 in synoviocytes. Synoviocytes characterized by qRT-PCR. Each *bar* shows the average ± standard deviation of three independent experiments from different patients (*n* = 5); **P* < 0.05. (DOCX 25 kb) Additional file 5: Characterization of cell populations. Synoviocytes culture marked with anti-CD166 and anti-CD14. Each *bar* shows the average ± standard deviation of three independent experiments from different patients (*n* = 5); **P* < 0.05. (DOCX 26 kb)

## Abbreviations

DNPH: 2, 4-dinitrophenylhydrazine
FC: flow cytometry
FLS: fibroblast-like synoviocytes
IL: interleukin
MP: misfolded glycoproteins aggregates
MSU: monosodium urate
NLRP3: NACHT, LRR and PYD domain-containing protein 3
NO: nitric oxide
OS: oxidative stress
RE: rough endoplasmic reticulum
RNS: nitrogen species
ROS: reactive oxygen species
SEM: scanning electron microscopy
SM: synovial membrane
TNF: tumor necrosis factor
UA: uric acid
WB: western blot
