# Supplementary material for: Monosodium urate crystals induce oxidative stress in human synoviocytes
Source: Arthritis Res Ther. 2016 May 21;18:117. doi: 10.1186/s13075-016-1012-3 (PMC4875700; doi:10.1186/s13075-016-1012-3)
Supplement: Additional file 1: Table S1. — Primers used for qRT-PCR gene analysis. (DOCX 14 kb) [file 13075_2016_1012_MOESM1_ESM.docx]

Additional file 1: Table 1. Primers used for qRT-PCR gene analysis.

| **Gene** | **ID** | **Catalogue** | **Annealing temperature** | **Melting** | **Product size** |
| --- | --- | --- | --- | --- | --- |
| **UGDH** | ID: 7358 | PPH08781A | 60°C | 79.75 °C | 90 pb |
| **CD14** | ID: 929 | PPH05723A | 60°C | 85.15°C | 152 pb |
| **GAPDH** | ID: 2597 | QT00079247 | 60 °C | 81.7 °C | 143 pb |
