# Supplementary material for: Monosodium urate crystals induce oxidative stress in human synoviocytes
Source: Arthritis Res Ther. 2016 May 21;18:117. doi: 10.1186/s13075-016-1012-3 (PMC4875700; doi:10.1186/s13075-016-1012-3)
Supplement: Additional file 2: — Synthetic crystals of monosodium urate. A Scanning electron micrograph of synthetic MSU crystals (×50,000). B MSU crystals under polarized light (×400). C MSU crystals from the synovial fluid of a gout patient (×400). The white bar indicates the axis compensator. (DOCX 1750 kb) [file 13075_2016_1012_MOESM2_ESM.docx]

Additional file 2: Synthetic crystals of monosodium urate. A) Scanning electron micrograph of synthetic MSU crystals (50,000 ×). B) MSU crystals under polarized light (400 ×). C) MSU crystals from the synovial fluid of a gout patient (400 ×). The white bar indicates the axis compensator.

**
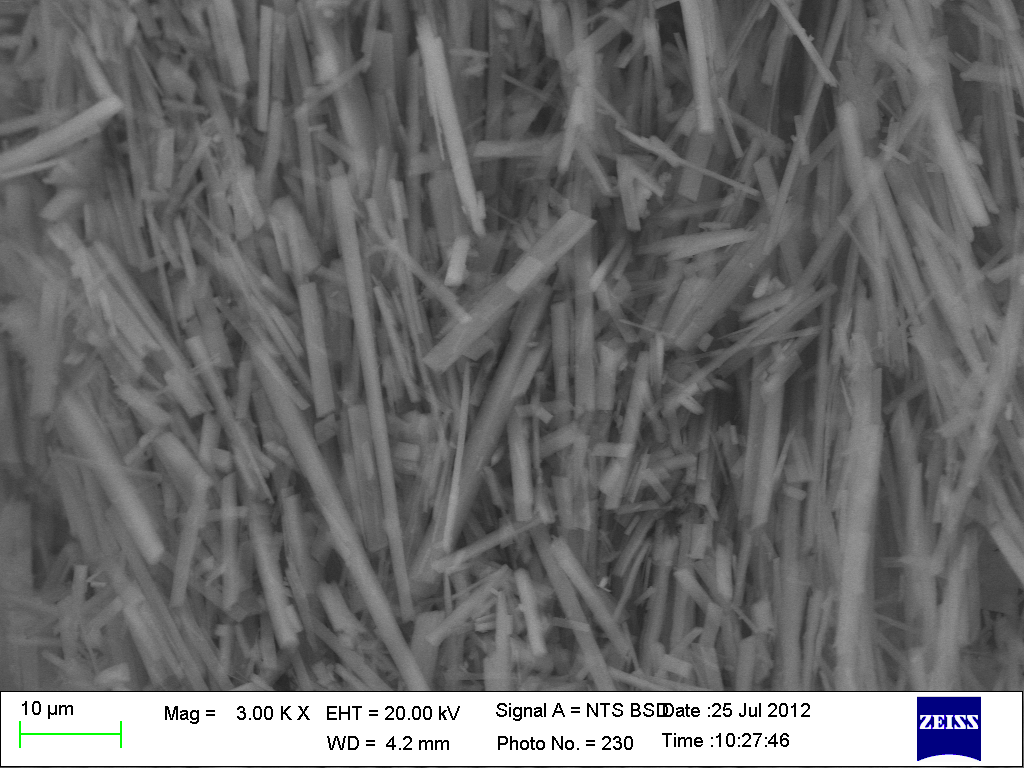
**


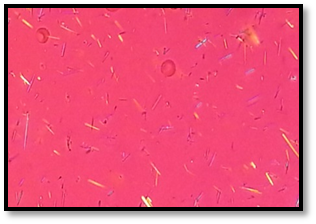

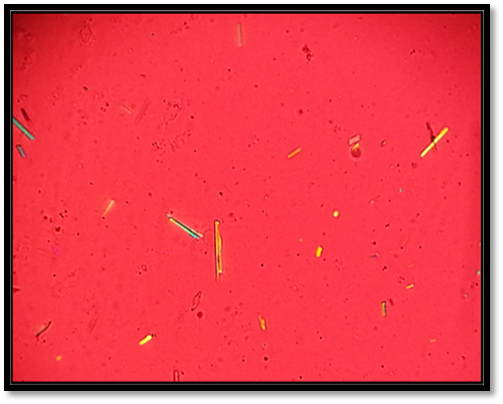


**C**

**B**

**A**
