# Supplementary material for: Monosodium urate crystals induce oxidative stress in human synoviocytes
Source: Arthritis Res Ther. 2016 May 21;18:117. doi: 10.1186/s13075-016-1012-3 (PMC4875700; doi:10.1186/s13075-016-1012-3)
Supplement: Additional file 3: — Characterization of synoviocytes derived from five primary cultures from different patients. A Typical morphology of fibroblasts. B Expression of PDH4+ in synoviocytes by IFA, expression of intracellularly localized PDH4+ by confocal microscopy. C Protein expression of PDH4+ by WB. (DOCX 840 kb) [file 13075_2016_1012_MOESM3_ESM.docx]

Additional file 3: Characterization of synoviocytes derived from five primary cultures from different patients. A) Typical morphology of fibroblasts. B) Expression of PDH4+ in synoviocytes by IFA, expression of intracellularly localized PDH4+ by confocal microscopy. C) Protein expression of PDH4+ by WB. Results are representative of one of five separate experiments.


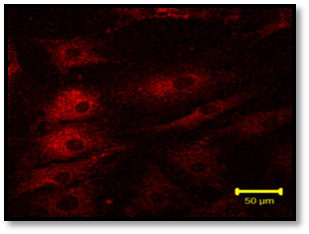

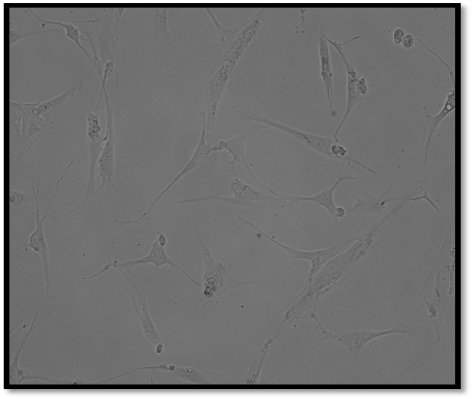

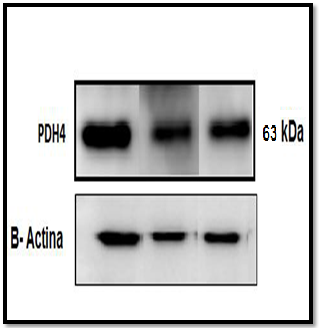


**A**

**B**

**C**
