# Supplementary material for: Monosodium urate crystals induce oxidative stress in human synoviocytes
Source: Arthritis Res Ther. 2016 May 21;18:117. doi: 10.1186/s13075-016-1012-3 (PMC4875700; doi:10.1186/s13075-016-1012-3)

Additional file 4: UGDH gene expression and CD14 in synoviocytes. Synoviocytes characterized by qRT-PCR. Each bar shows the average ± standard deviation of three independent experiments from different patients (n = 5) **P*<0.05.


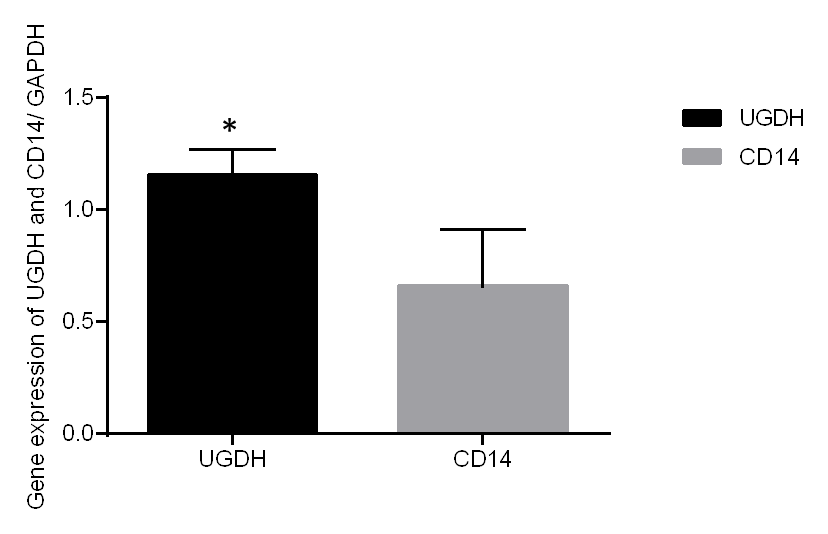

Supplement: Additional file 4: — UGDH gene expression and CD14 in synoviocytes. Synoviocytes characterized by qRT-PCR. Each bar shows the average ± standard deviation of three independent experiments from different patients (n = 5); *P < 0.05. (DOCX 25 kb) [file 13075_2016_1012_MOESM4_ESM.docx]
